# A Low Concentration of a Sustainably Obtained Blueberry Extract Improves the Post‐Thawing Motility of Cryopreserved Bull Spermatozoa

**DOI:** 10.1111/rda.70238

**Published:** 2026-09-30

**Authors:** Gabriela García‐Blanco, Cristina Fra‐Hernández, Joana F. do‐Vale‐Rabaça, Laura Pariente‐Martín, Marta Veza‐Cuenca, Estela Fernández‐Alegre, Beatriz Martín‐Fernández, J. Néstor Caamaño, J. Ramiro González‐Montaña, Marta Lores, Felipe Martínez‐Pastor

**Affiliations:** ^1^ INDEGSAL, Universidad de León León Spain; ^2^ Department of Molecular Biology (Cell Biology) Universidad de León León Spain; ^3^ IMAPOR, Universidad de León León Spain; ^4^ Bianor Biotech SL León Spain; ^5^ Selección y Reproducción Animal‐SERIDA, Principado de Asturias Gijón Spain; ^6^ Department of Medicine, Surgery and Veterinary Anatomy Universidad de León León Spain; ^7^ Department of Analytical Chemistry, Nutrition and Food Science, Faculty of Chemistry, LIDSA Universidade de Santiago de Compostela Santiago de Compostela Spain

**Keywords:** antioxidants, blueberry, bull, cryopreservation, natural extracts, sperm motility

## Abstract

Natural extracts could improve sperm storage and artificial insemination (AI). This study, for the first time, evaluates the suitability of a blueberry extract ( *Vaccinium corymbosum* ), obtained from pomace using a sustainable methodology, as a supplement for bull semen extenders. Cryopreserved semen doses from eight bulls were combined in 9 pools (3 bulls/pool), supplemented with 0%, 1%, 5%, or 10% extract and incubated up to 5 h at 38°C. Motility was assessed hourly using OpenCASA, and the effects of treatment and time were evaluated using linear mixed‐effects models. Motility was significantly better preserved with 1% extract (total and progressive motility, linear velocity and linearities, with lower BCF and fractal dimension, related to hyperactivation). The effect of 5% was overall positive but lower than 1%, whereas 10% showed a mostly negative effect. This natural extract could safely supplement bull semen extenders by at least 5% and even help improve sperm motility. Therefore, its use as a supplement in bull semen extenders offers an opportunity to enhance assisted reproductive techniques in cattle using a sustainable approach.

## 1 Introduction

The interest in applying natural extracts to semen extenders has increased notably (Ros‐Santaella and Pintus 2021; Schulze et al. 2020). With complex compositions, these extracts are rich in antioxidant and antimicrobial molecules, such as polyphenols (Gato et al. 2021). These properties are especially attractive for sperm cryopreservation, a stressful process in which redox imbalance can decrease sperm fertility. Moreover, the antimicrobial properties could help reduce antibiotic use (Elmi et al. 2019). These applications are extremely important in the cattle industry, where sperm cryopreservation and artificial insemination (AI) are widespread.

There is ample literature on the use of polyphenols and natural extracts (Ros‐Santaella and Pintus 2021; Santos and Silva 2020). However, there is a lack of studies on the application of extracts obtained from agricultural waste or by‐products. These extracts are promising not only because they are of natural origin and free of synthetic substances, but also because they contribute to the circular economy concept and to the revalorisation of refuse (marc or pomace).

Thus, we explored the application of a blueberry ( *Vaccinium corymbosum* ) pomace extract as a supplement to bull semen extenders. Since polyphenols and other substances may be detrimental, this pilot experiment assessed increasing concentrations on the motility of post‐thawed bull semen. As an added novelty and benefit, the extract was produced using an improved ‘green’, environmentally‐friendly methodology (Lončarić et al. 2020).

## 2 Materials and Methods

Semen was collected from eight adult, fertile Holstein bulls using an artificial vagina (Xenética Fontao, Lugo, Spain), extended with the same volume of BoviFree (Minitüb, Tiefenbach, Germany), cooled to 4°C and donated to the researchers. Ejaculates were extended to 92 × 10^6^/ml in BoviFree, equilibrated, packaged into 0.25‐mL straws and frozen in an IceCube 1800 biofreezer (SY‐LAB, Neupurkersdorf, Austria): −5°C/min to −10°C; −40°C/min to −100°C; −20°C/min to −140°C. Doses were stored in liquid nitrogen. In each experimental session, three straws from different bulls were thawed (water bath at 37°C, 30 s) and mixed into a pool, split and supplemented with 0% (control), 1%, 5%, or 10% aqueous blueberry pomace extract (LIDSA, University of Santiago de Compostela, Spain); dilutions were planned so all treatments contained the same final volume of saline vehicle (pH and osmolality not differing among treatments). The samples were incubated at 38°C up to 5 h.

Motility was assessed hourly with a CASA‐mot system (Tables S2 and S3) using the OpenCASA v. 2 software (Alquézar‐Baeta et al. 2019) to obtain total and progressive motility, velocity (VCL, VAP, VSL), linearity (LIN, STR, WOB), ALH, BCF, mean Dance (mDNC) and the fractal dimension (the degree to which the sperm trajectory fills a plane, associated with hyperactivation; Boryshpolets et al. 2015).

The experiment consisted of nine pools. Data were analysed by linear mixed‐effects models (R statistical environment v. 4.2), with treatment and incubation time as fixed factors and bull in the random part of the models.

## 3 Results and Discussion

Our results support the use of this blueberry extract as a supplement for bull semen extenders. Figures 1 and 2 summarise each treatment along the incubation time (details in Table S1). After adding 1% of the extract, total motility (Figure 1a) improved over the control throughout the incubation (12.7 ± 1.0 overall increase); progressivity, velocity and linearity parameters (Figures 1b,c,f and 2a,b) increased similarly (e.g., overall effect of 7.6 ± 1.3 for VSL and 5.0 ± 0.7 for LIN). We also detected significantly lower fractal dimension (Figure 2f) and BCF (beat‐cross frequency, Figure 2e). Thus, supplementation with a low concentration could improve post‐thawing motility and resilience in bull sperm.

**FIGURE 1:**
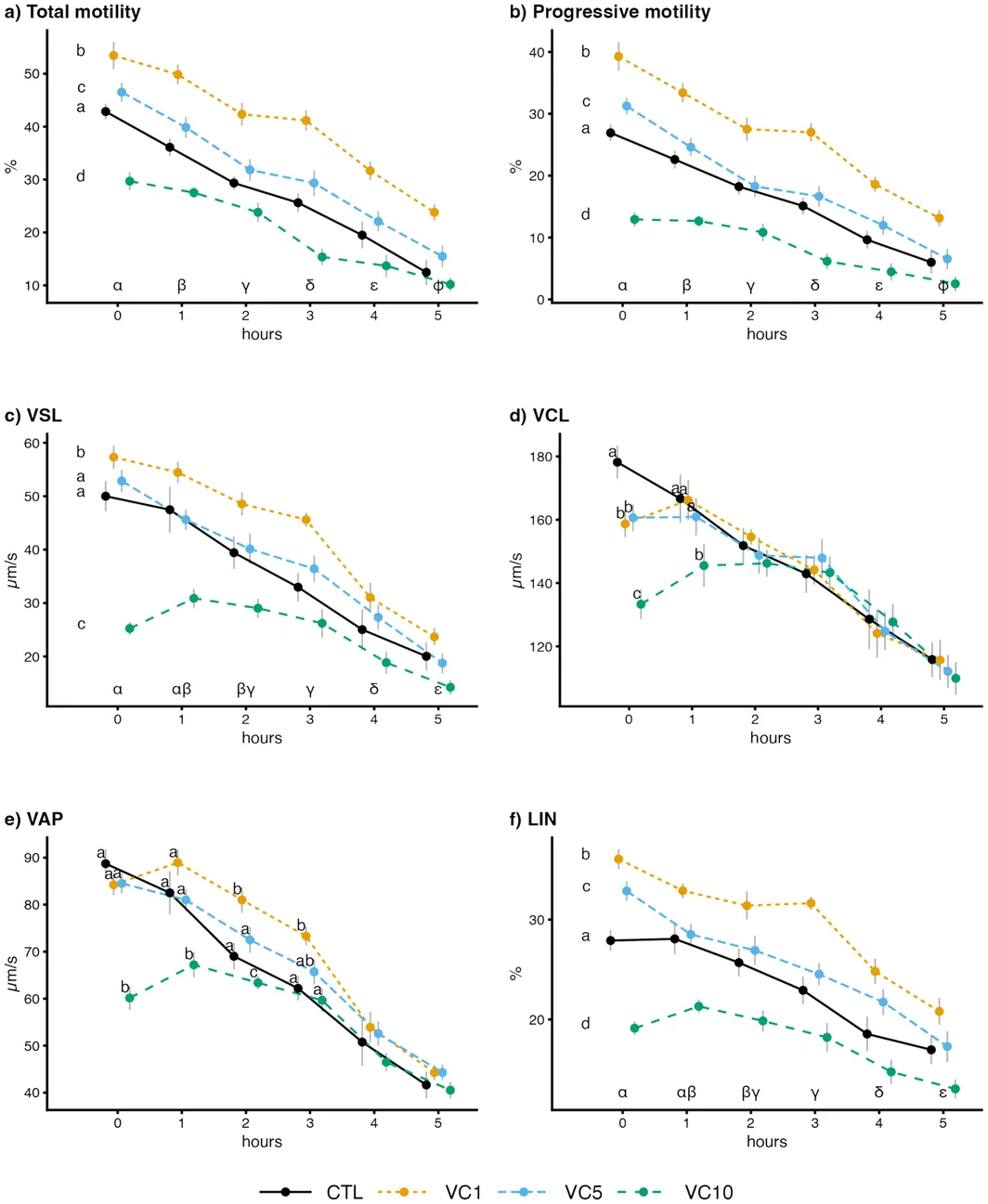
Sperm motility results (mean ± SEM, *n* = 9); parameter description in Table S4 in the supporting information. For total motility, progressive motility, VSL and LIN, the treatment × time interaction was not significant, and the comparison was carried out on main effects (Holms's adjustment; Latin letters on the left indicate treatment differences and Greek letters indicate time differences). For VCL and VAP, the interaction was significant, with letters indicating differences among treatments at each time point (to prevent cluttering, differences among times are shown in the Suppl. Mat., Table S1). Treatments are CTL: control; VC1: 1% extract; VC5: 5% extract; VC10: 10% extract.

**FIGURE 2:**
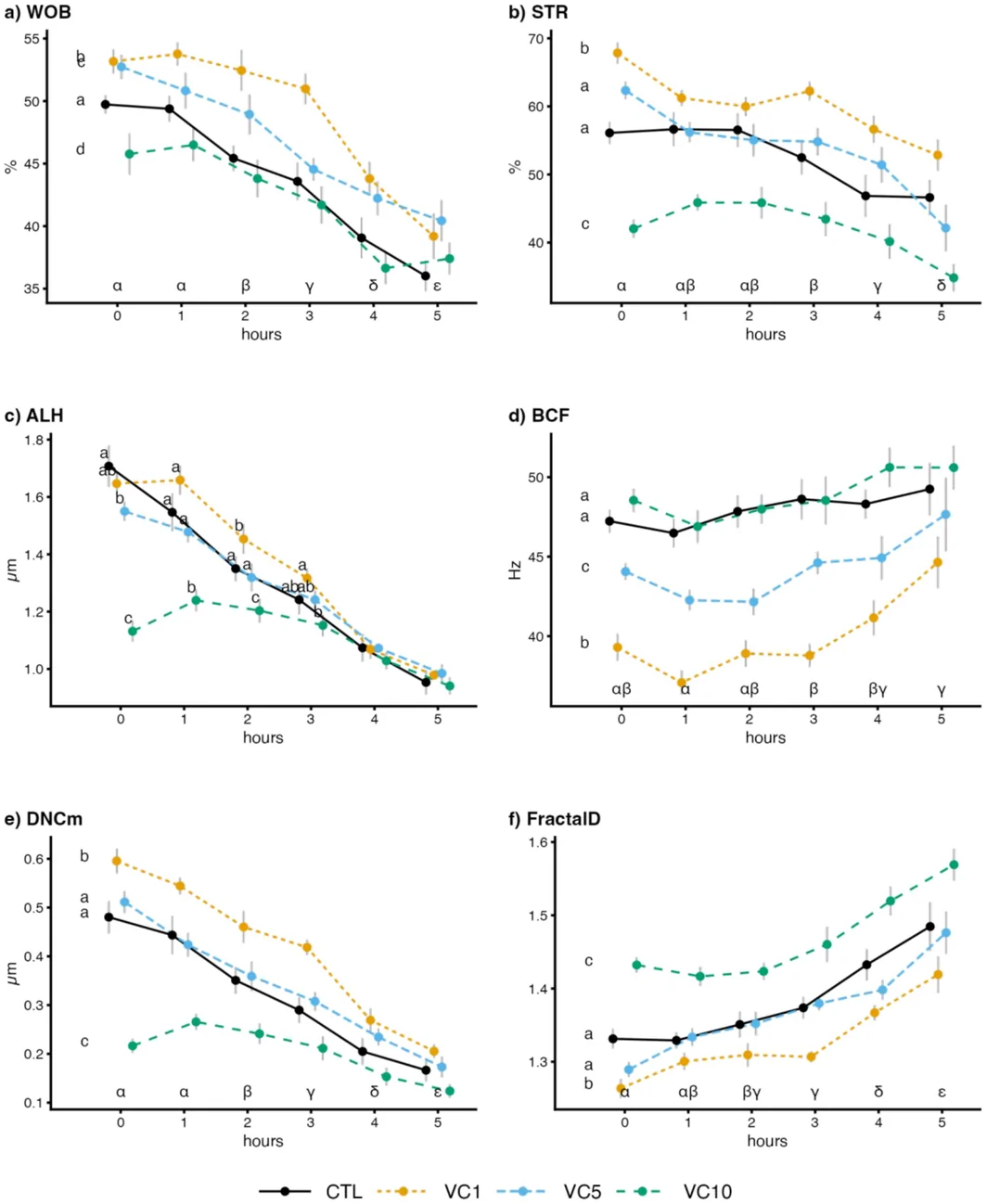
Sperm motility results, see description in Figure 1 (significant interaction only for ALH). See Table S4 for parameter description and Table S1 for extended results in the supporting information.

At higher concentrations, we observed a positive effect with 5% extract (*p* < 0.05 in most cases), although the effect was much smaller than at 1%. The complex composition of natural extracts implies a mixture of stimulant and inhibitory chemicals, whose balance could be less favourable at higher concentrations. Indeed, a physiological level of oxidative species is critical for adequate intracellular signalling (Das and Roychoudhury 2022), and it could be jeopardised by excessive antioxidants. The motility depression and non‐linearity observed in the 10% treatment support this interpretation. Nevertheless, mechanistic associations are at this point speculative and must be tested in extended studies.

A major objective of this study was to define safe concentrations for a continuing study of the extract's applications. Natural extracts can exhibit cytotoxicity; therefore, a first definition of a safe range is critical for the development of new formulations. A limitation of this pilot study is that only motility was considered. Subsequent studies should assess sperm integrity, metabolism, capacitation markers and chromatin status to elucidate the extract's effects and better define an optimal concentration.

The complex composition of natural extracts might complicate their use and marketability; however, this complexity could be an advantage, as when used as antimicrobials (Gato et al. 2021). In this regard, this blueberry pomace extract contains a wide range of polyphenols and other molecules, many of which are effective antioxidants (Lončarić et al. 2020). Likewise, these natural chemicals modulate membrane pumps and metabolism (Nass‐Arden and Breitbart 1990) and possibly intracellular pathways. Our experiment was limited, and therefore, we cannot claim a causal relationship. Thus, a hypothesis remains to be tested: whether specific polyphenols or the overall antioxidant properties are responsible for the motility changes observed across the different treatments.

Nevertheless, the results obtained with only 1% offer an excellent prospect for future work, not only for post‐thawing improvement but potentially for refrigerated and cryopreserved storage. Moreover, bull spermatozoa exhibited good tolerance to a relatively high concentration of this extract (5%), indicating promise for exploiting its potential antimicrobial properties (Gato et al. 2021).

In conclusion, the blueberry pomace extract tested in this study showed promising protective effects at only 1% in post‐thawed bull semen, with smaller but non‐negative effects at 5%. This extract could be useful for enhancing semen extenders in this species, for sperm cryopreservation, and maybe for antibiotic substitution, given its antimicrobial properties; however, these effects were not considered in the present study and remain untested. Finally, the natural and environmentally friendly origin of this extract might contribute to a more sustainable animal production sector.

## Author Contributions

G.G.B. designed the experiment, carried out the experiments and analysed the data. C.F.‐H., J.F.V.R., L.P.‐M., M.V.‐C. and E.F.‐A. carried out the experiments and analysed the data. B.M.‐F., J.N.C. and J.R.G.‐M. edited the manuscript. M.L. carried out the experiments and edited the manuscript. F.M.‐P. coordinated the study, coordinated the data analysis and drafted the manuscript. All authors read and approved the final manuscript.

## Funding

This work was supported by the grant PID2022‐137640OB‐I00 (supported by MCIN/AEI/10.13039/501100011033 and ‘ERDF A way of making Europe’). M.V.‐C. and L.P.‐M. were supported by the University of León and Junta de Castilla y León/ESF+ (EU), respectively.

## Conflicts of Interest

The authors declare no conflicts of interest.

## Supporting information


**Table S1:** Provides summary results and complete statistical significances. The supplementary document includes details regarding the CASA system, Tables S2 and S3 detailing the configuration for image acquisition and analysis, and Table S4 describing CASA parameters.

**Table S2:** Configuration of the Basler Pylon software for acquiring bull sperm motility videos.
**Table S3:** Configuration of the OpenCASA v. 2 software for analysing the bull sperm motility AVI videos.
**Table S4:** Description of the CASA parameters (definitions taken from Boyers et al. 1989; fractal dimension from Alquézar‐Baeta et al. 2019, Mortimer et al. 1996).

## Data Availability

The data supporting the findings of this study are openly available on Zenodo at https://doi.org/10.5281/zenodo.19359494.
